# Hydrothermal and Organosolv Treatments for Hydroxycinnamate Release from Corn Stover: Strong *versus* Mild Alkaline Catalysis

**DOI:** 10.3390/molecules30214297

**Published:** 2025-11-05

**Authors:** Evangelia Brimo-Alevra, Marina Koutli, Elli Marielou, Theodoros Chatzimitakos, Dimitris P. Makris

**Affiliations:** Green Processes & Biorefinery Group, Department of Food Science & Nutrition, School of Agricultural Sciences, University of Thessaly, N. Temponera Street, 43100 Karditsa, Greece

**Keywords:** antioxidants, polyphenols, ferulic acid, *p*-coumaric acid, corn stover treatment, agri-food waste valorization

## Abstract

Corn stover (CS) is an abundant biomaterial, which is regularly rejected during corn harvesting. This biowaste is a typical lignocellulosic source rich in hydroxycinnamates, which are mainly represented by *p*-coumaric acid and ferulic acid. These polyphenols are largely bound onto the lignocellulosic complex and can be effectively liberated using alkaline catalysis. On this basis, the work described herein targeted at developing a high-performance process for producing hydroxycinnamate-enriched extracts, by deploying alkali-catalyzed hydrothermal and organosolv treatments. For this purpose, sodium carbonate was tested as a benign, natural alkali catalyst, along with the well-studied sodium hydroxide. The kinetic study demonstrated that both the alkali catalyst and the organic solvent (ethanol) may significantly affect polyphenol recovery, a fact further investigated by carrying out response surface optimization. The hydrothermal treatment was shown to be more efficacious than the organosolv one, with regard to total polyphenol recovery, while the sodium carbonate catalysis was less efficient compared to the sodium hydroxide one. Under optimized conditions, the hydrothermal treatment afforded 74.4 ± 3.6 mg gallic acid equivalents per g of dry CS mass. On the other hand, a more thorough investigation of the polyphenolic profile of the extracts obtained clearly demonstrated that the sodium hydroxide-catalyzed organosolv treatment provided almost 76 and 98% higher yields for *p*-coumaric and ferulic acid, respectively, compared to the hydrothermal treatment. Extract composition impacted the antioxidant activity, and it was revealed that the higher the *p*-coumaric acid/ferulic acid ratio, the stronger the antioxidant effect. It is proposed that the sodium hydroxide-catalyzed ethanol organosolv treatment of CS may be a particularly promising technique in a lignocellulose biorefinery frame, although improvements might be necessary to further increase treatment performance. Such a process might contribute to fully valorizing agricultural biowastes for the production of high value-added chemicals, in line with the “lignin first’ philosophy.

## 1. Introduction

The most abundant renewable detritus is lignocellulosic biomass, with a global production estimated to reach about 182 billion tons on an annual basis; of this amount, only around 8 billion tons are currently exploited. Lignocellulosic biomass includes mainly wood processing residues and agri-food biowastes, which contains three of the most abundant natural biopolymers, namely cellulose, lignin, and hemicellulose [1,2,3,4]. Compared to other biopolymers, lignin is a particularly intricate macromolecule, and its content may range between 15% and 40% *w*/*w* in woody plants and from 12% to 21% *w*/*w* in straw species, on a dry weight basis [5]. Lignin is a primal recalcitrant component in lignocellulosic matrices, and it largely defines biomass utilization technologies for value-added products, such as phenolic compounds. Thus, by virtue of its high abundance and low cost, lignin may have a significant perspective in biorefinery strategies for producing polyphenolic substances [6].

Corn (*Zea mays*) is one of the major cereal crops, used globally to produce food and feed, as well as other industrially important products (e.g., alcohol, cornstarch, etc.). The world corn production was mounted to about 1.03 billion tons in 2017 [7], and in Greece, the mean corn production per year for the period of 2002–2020 was estimated to be around 2042 thousand tons [8]. The harvesting and processing of corn generates a high volume of side streams, the major being the corn stover (CS). This residual biomass comprises husks, stalks, and leaves that are left in the field after harvesting, and it is generated at a level of 1 dry kg per dry kg of corn grain [9]. Therefore, CS production mounts globally up to almost 1 billion tons.

One of the main biopolymers occurring in CS is lignin, its content being around 12% *w*/*w*. Hence, CS is regarded as a major lignocellulosic material for the production of high value-added chemicals, such as building block molecules and lignin-associated polyphenolic antioxidants [7]. Lignin-derived hydroxycinnamates in CS embrace large amounts of pendant units, linked onto lignin primarily through both ether and ester bonds (∼18% *p*-coumaric acid on a lignin basis) [10], and the major hydroxycinnamate antioxidants in various CS tissues are ferulic acid and *p*-coumaric acid [11]. These components may serve as cross-linking bridges between polysaccharide chains and proteins and lignin, but they can also cross-couple with monolignols and be incorporated as co-polymers into lignin networks [12].

Due to their multifaceted biological properties, hydroxycinnamates are regarded as natural substances of nutritional and/or pharmaceutical importance, and as widespread dietary antioxidants. The antioxidant potency of major hydroxycinnamates has been well substantiated, and several studies have been performed to document structure–activity relationships [13,14,15]. The biological significance of hydroxycinnamates does not solely pertain to their antioxidant activity, since several beneficial effects on human health have been evidenced by an assortment of relevant examinations [16,17,18,19].

Bound polyphenol recovery from cereal tissues demands treatments that involve alkaline and/or acid catalysis, while polyphenol species released from such processes may be largely affected by the type of catalyst (alkaline/acid) employed. Therefore, a number of different hydroxycinnamates and their derivatives have been identified in maize extracts, produced by using alkaline or acid catalysis and variable processing conditions [20]. Recent data evidenced that sodium hydroxide-catalyzed ethanol organosolv treatments of wheat bran may produce extracts highly enriched in ferulic acid [21], yet the testing of mild catalysts, such as citric acid and sodium carbonate, showed that such catalysts may also be very effective in releasing ferulates in a hydrothermal treatment. However, in this case it was demonstrated that the alkali-catalyzed treatment afforded substantially diversified composition than the acid-catalyzed one [22].

Considering the above findings, this work was undertaken to examine the potency of hydrothermal and ethanol organosolv treatments to recover hydroxycinnamates from CS. In both cases, a strong (sodium hydroxide) and a mild (sodium carbonate) catalyst were compared to investigate their efficiency in the treatment performance. As far as the authors are concerned, such an approach for effective polyphenol recovery from CS is addressed for the first time.

## 2. Results and Discussion

### 2.1. Effect of Alkali Catalyst Concentration on the Hydrothermal Treatment

**Figure 1:**
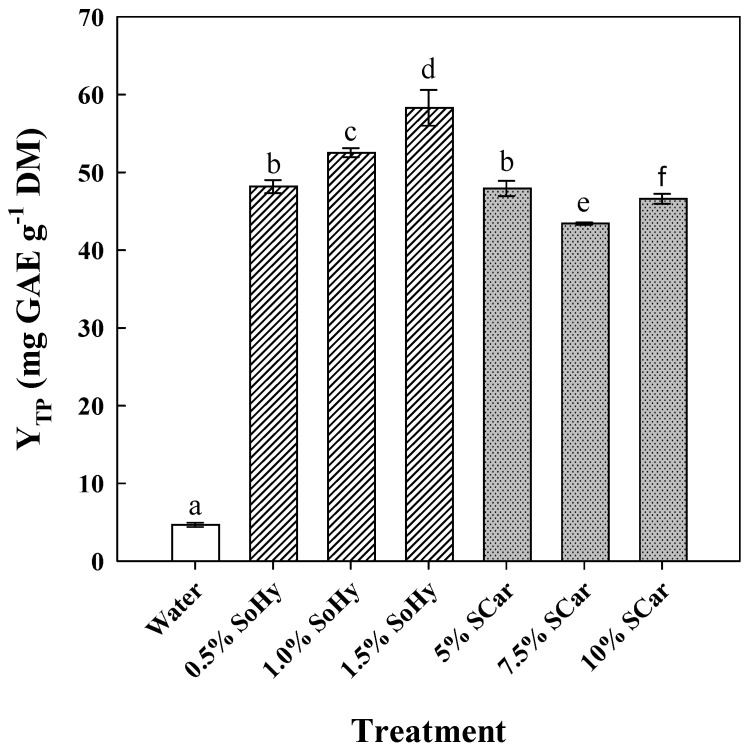
The effect of sodium hydroxide (SoHy) and sodium carbonate (SCar) concentration on the total polyphenol yield (Y_TP_), upon hydrothermal treatment of corn stover at 90 °C, for 300 min. Bars represent means (*n* = 3) ± s.d. Bars designated with different letters (a, b, c, d, e, and f) represent statistically different values (*p* < 0.05).

The role of the alkali catalyst in boosting polyphenol recovery from CS was assessed by using a mild (sodium carbonate—SCar) and a strong (sodium hydroxide—SoHy) catalyst, over concentration ranges selected according to recent investigations [21,22]. Thus, SoHy was tested within 0.5 to 1.5% (*w*/*v*) (pH range 12.81–13.23), while SCar within 5 to 10% (*w*/*v*) (pH range 11.48–11.76) (Figure 1). Compared to the treatment carried out with neat water, which afforded a Y_TP_ of 4.7 mg GAE g^−1^ DM, treatment with 1.5% SoHy was found to be the most effective (*p* < 0.05), giving almost 12.5 times higher Y_TP_ (58.3 mg GAE g^−1^ DM). The use of 5% SCar also boosted polyphenol recovery approximately 10 times, but further increase in SCar concentration did not favor increase in Y_TP_.

**Figure 2:**
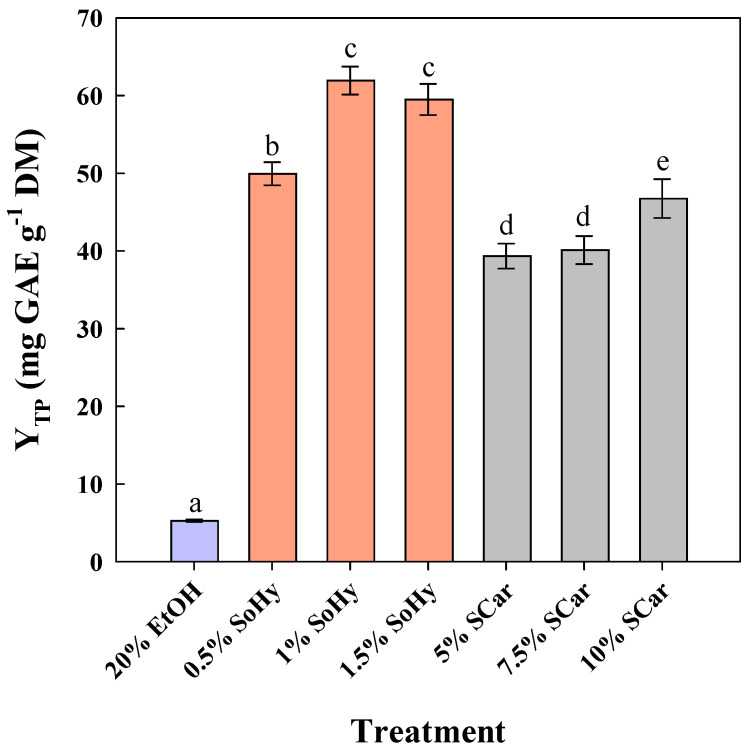
The effect of sodium hydroxide (SoHy) and sodium carbonate (SCar) concentration on the total polyphenol yield (Y_TP_), upon organosolv treatment (20% ethanol) of corn stover at 80 °C, for 300 min. Bars represent means (*n* = 3) ± s.d. Bars designated with different letters (a, b, c, d, and e) represent statistically different values (*p* < 0.05).

To examine the effect of ethanol, 20% aqueous ethanol mixtures were employed, and both alkali catalysts were assayed again within the same concentration ranges (Figure 2). The level of 20% ethanol was chosen considering the limitation arising from SCar solubility [23].

Treatment of CS using 20% ethanol (no catalyst added) gave a Y_TP_ of 5.3 mg GAE g^−1^ DM, while the addition of 1% SoHy greatly buttressed treatment performance, affording around 12 times higher Y_TP_ (62.0 mg GAE g^−1^ DM). However, unlike the hydrothermal treatment, switching SoHy concentration from 1 to 1.5% did not increase Y_TP_, but a non-significant decline was observed (*p* > 0.05). On the contrary, the best performance using SCar as catalysts was seen at a concentration of 10%, where a Y_TP_ of 46.8 mg GAE g^−1^ DM was attained. This outcome showed that the effect of both catalysts may be significantly differentiated by the presence of ethanol.

### 2.2. Kinetics of Polyphenol Recovery

**Figure 3:**
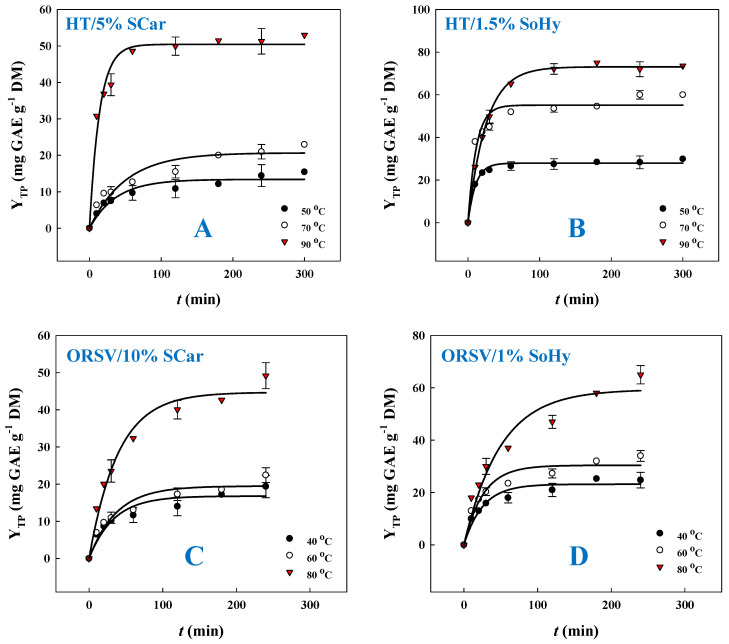
Polyphenol release kinetics from corn stover, upon hydrothermal treatment with 5% sodium carbonate (**A**) and 1.5% sodium hydroxide (**B**), and organosolv (20% ethanol) treatment with 10% sodium carbonate (**C**) and 1% sodium hydroxide (**D**).

Preliminary experimentation was carried out to choose an appropriate kinetic model, and, on this ground, a first-order model was found to provide the highest suitability. Thus, polyphenol release kinetics was monitored using the first-order model, and the outcome of this assay is portrayed in Figure 3 and Table 1. To better portray the effect of the type of alkali catalyst and the role of the solvent (ethanol) in the polyphenol recovery from CS, temperatures varying from 50 to 90 °C were used to carry out kinetics. Based on a first-order model, the extraction rate constant, *k*, and the total polyphenol yield at saturation, Y_TP(*s*)_, could be estimated (Table 1). For the SoHy-catalyzed hydrothermal treatment, the maximum extraction rate *k* (93.9 × 10^−3^ min^−1^) was attained at 50 °C, whereas a decrease in *k* was seen when temperature was switched from 50 to 90 °C. However, maximum Y_TP(*s*)_ (73.1 mg GAE g^−1^ DM) was reached at 90 °C. On the contrary, for the SCar-catalyzed hydrothermal treatment, both maximum *k* (70.9 × 10^−3^ min^−1^) and maximum Y_TP(*s*)_ (50.4 mg GAE g^−1^ DM) were recorded at 90 °C. This outcome revealed that the SoHy-catalyzed treatment was far more effective, in terms of achieving a high Y_TP(*s*)_.

On the other hand, the SoHy-catalyzed organosolv treatment displayed much lower *k* values compared to the corresponding hydrothermal one, irrespective of the temperature tested. Furthermore, the maximum Y_TP(*s*)_ (59.4 mg GAE g^−1^ DM), achieved at 80 °C, was almost 19% lower than that achieved with the hydrothermal treatment. Likewise, the SCar-catalyzed organosolv treatment displayed a significantly lower *k* at 80 °C compared to the hydrothermal one at 90 °C, while the maximum Y_TP(*s*)_ in this case was about 11% lower than that obtained with hydrothermal treatment. On such grounds, it was clear that the presence of ethanol modified kinetics in a non-consistent fashion, but hydrothermal treatment was more efficacious for polyphenol recovery, irrespective of the catalyst used.

### 2.3. Optimization of Treatments Through Response Surface Methodology

The treatments proven to be the highest-performing processes, that is, the hydrothermal treatments with 1.5% SoHy and 5% SCar, and the organosolv treatments with 1% SoHy and 10% SCar, were optimized by deploying response surface methodology. The optimization was designed to assess the effect of the two key treatment variables (*t*, *T*), but also to reveal any synergistic functions between these variables. Evaluation of the fitted model and the suitability of the response surface methodology used were made on the basis of analysis of variance (ANOVA) and lack-of-fit test (Figures S1–S4), taking into account measured and predicted values proximity (Tables S1–S4). The mathematical models (second-degree polynomial equations) derived from the response surface optimization are given in Table 2.

**Figure 4:**
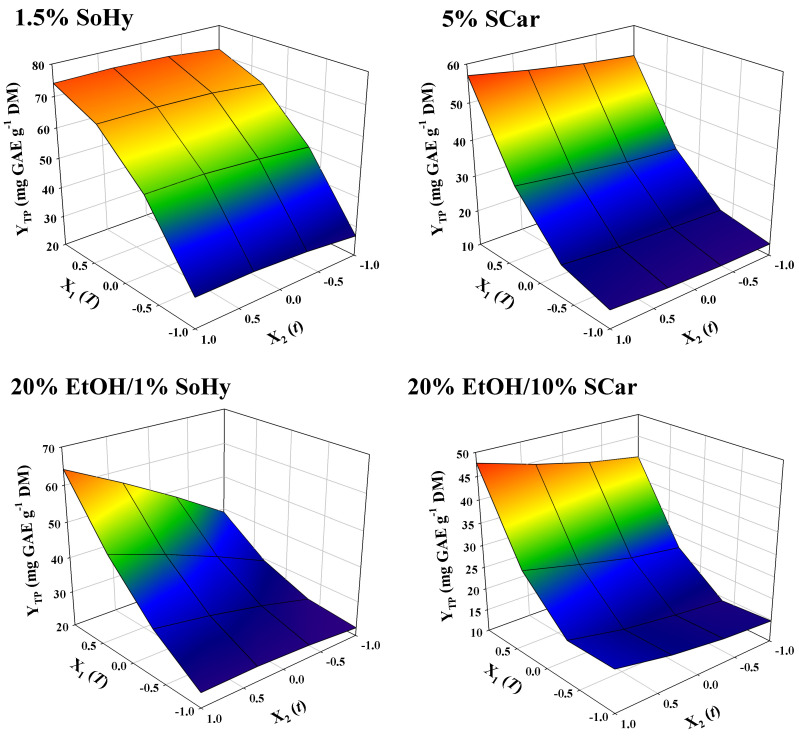
Representation of the simultaneous effect of treatment variables (*t*, *T*) on the total polyphenol yield (Y_TP_) by three-dimensional graphs.

The models contained only significant terms, whereas non-significant ones were omitted. The square correlations coefficients (R^2^) determined for each model provided an account of the total variability around the mean, given by the model. It can be seen in Table 2 that R^2^ ≥ 0.97 for all models, and the *p* value for lack-of-fit (based on a 95% confidence interval), was highly significant. On such a ground, it could be supported that the models displayed excellent adjustment to the experimental data. An at-a-glance depiction of the effect of independent variables on the response, given as 3-dimentional diagrams, may be seen in Figure 4. For the SoHy-catalyzed hydrothermal treatment, time within the limits tested had non-significant influence (*p* > 0.05), whereas temperature had a significant impact on Y_TP_, obeying a quadratic function (Table 2). By contrast, for both the hydrothermal and organosolv treatments catalyzed by SCar, *t* was a significant factor. Furthermore, for the SoHy-catalyzed organosolv treatment it was found that cross effects of the two independent variables (X_1_X_2_) also had a positive and significant effect on Y_TP_. To determine the optimum values for both variables (*T*, *t*) but also the maximum response value (Y_TP_), the desirability function was employed (Figures S1–S4), and the results obtained are analytically displayed in Table 3.

The SoHy-catalyzed hydrothermal treatment provided a maximum Y_TP_ of 74.4 ± 3.6 mg GAE g^−1^ DM, which did not show statistically significant difference (*p* > 0.05) from the Y_TP_ achieved with SoHy-catalyzed organosolv treatment. On the contrary, the SCar-catalyzed hydrothermal treatment afforded Y_TP_ of 57.1 ± 3.7 mg GAE g^−1^ DM, which was significantly higher (*p* < 0.05) than the Y_TP_ obtained with the organosolv treatment. Moreover, for each treatment, the SoHy catalysis was shown to be significantly more effective than the SCar catalysis in attaining high polyphenol recovery. To illustrate the effect of alkali catalysis on the recovery of polyphenol from CS, treatments with 20% ethanol and neat water were also performed, employing optimal *T* and *t* settings (90 °C/180 min and 80 °C/240 min, for the hydrothermal and organosolv treatments, respectively). The corresponding Y_TP_ values were 3.7 ± 0.1 and 5.3 ± 0.3 mg GAE g^−1^ DM, a finding that clearly demonstrated the pivotal role of the alkali catalysts in promoting polyphenol recovery.

Although in any case the examined SoHy catalysis was significantly more efficacious compared to the SCar catalysis, the potency of the latter should not be overlooked, as it may be regarded as a mild, non-corrosive alternative. Additionally, comparison between SCar-catalyzed hydrothermal and organosolv treatments revealed that the use of ethanol did not offer any advantage, since the organosolv treatment was proven less efficient than the corresponding hydrothermal ones. This finding contrasted previous results from studies on cotton stalk treatment, where the role of ethanol was critical in boosting SCar-catalyzed polyphenol recovery [23]. A critical role in ethanol has also been found for corncob treatment, in the non-catalyzed total polyphenol recovery [24].

To gain a deeper insight into the combined effect of *T* and *t*, but also to consider the effect of the treatment pH, the alternative combined severity factor (CSF′) was determined to appraise treatment severity (Table 3). The SoHy-catalyzed organosolv treatment had a CSF′ value of 8.19, which was higher compared to the 7.41 required for maximum Y_TP_ in wheat bran treatment, using 1.5% SoHy [21]. On the other hand, the value of 7.64 found for the SoHy-catalyzed organosolv treatment was comparable to 7.78, determined for maximum Y_TP_ from oat bran using 40% 1-propanol/1.5% SoHy [25], and 7.99 determined for maximum Y_TP_ from cotton stalks using 20% ethanol/0.5% SoHy [23]. Thus, it could be argued that the SoHy-catalyzed organosolv treatment used under optimized conditions may afford virtually equal polyphenol recovery with the SoHy-catalyzed hydrothermal one, requiring lower severity.

CSF′ is a measure to evaluate and optimize ethanol organosolv treatments [26], and it has been recently employed as a means of assessing process severity for polyphenol recovery [27]. However, it should be regarded as merely indicative, because the differences observed in various treatments might be ascribed to the recalcitrance of the treated material, but they may also be associated with the other attributes. For example, effective release of polysaccharide- and/or lignin-bound phenolics, such as those occurring in CS, may require higher temperatures, stronger alkaline conditions and longer residence time, with inevitably higher severity demands. Anyhow, it is of paramount importance to consider temperature, pH, and residence time [28] when designing a treatment for polyphenol recovery and, considering that severity may in some instances be highly correlated with recovery yield [21,29,30], then it might be employed as a useful tool in the appraisal of treatment performance.

### 2.4. Hydroxycinnamate Composition

**Figure 5:**
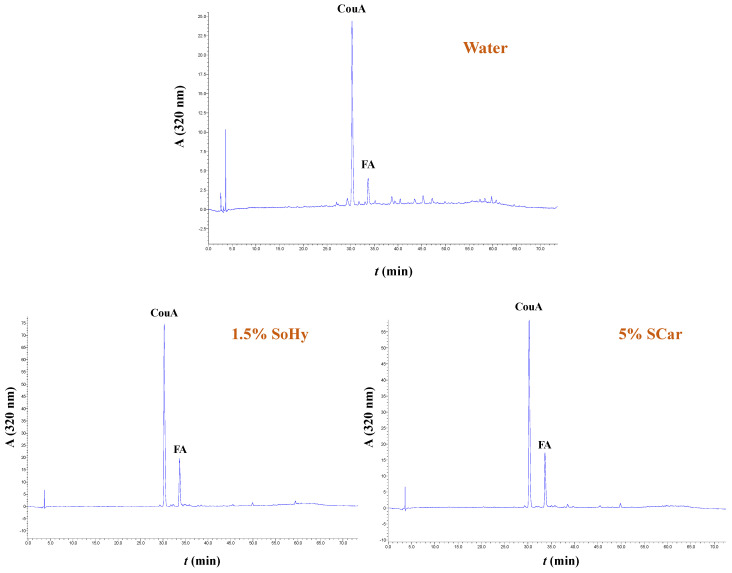
Polyphenol traces of CS extracts, obtained by deploying hydrothermal treatment. The chromatograms were monitored at 320 nm. Samples assigned as “Water”, “1.5% SoHy”, and “5% SCar” correspond to treatments performed with neat water, 1.5% sodium hydroxide, and 5% sodium carbonate.

**Figure 6:**
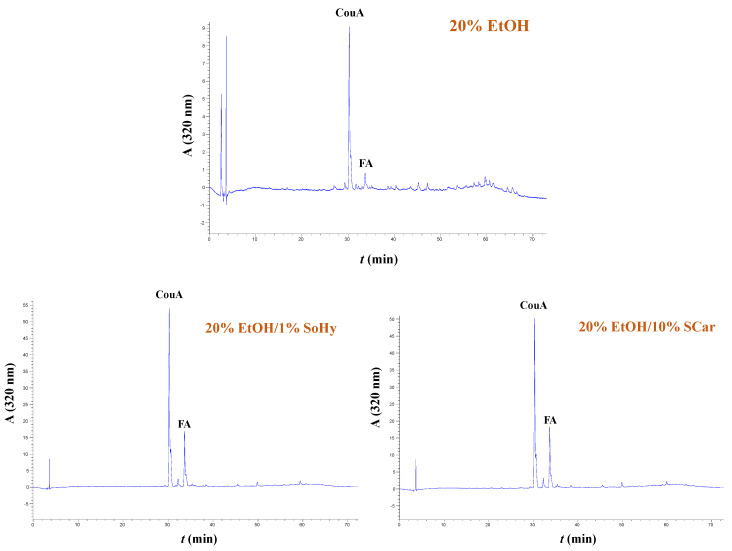
Polyphenol traces of CS extracts, obtained by deploying organosolv treatment. The chromatograms were monitored at 320 nm. Samples assigned as “20% EtOH”, “20% EtOH/1% SoHy”, and “20% EtOH/10% SCar” correspond to treatments performed with 20% ethanol, 20% ethanol/1% sodium hydroxide, and 20% ethanol/10% sodium carbonate.

The extracts generated from both the organosolv and hydrothermal treatments, and the control extracts produced with treatments performed with 20% ethanol and neat water, were analyzed by HPLC to shed more light into the effect of both the solvent and alkali catalyst on the polyphenolic composition. In all cases examined, it was shown that the extracts were predominated mainly by *p*-coumaric acid (CouA), accompanied by ferulic acid (FA), irrespective of the presence of ethanol or the alkali catalyst used (Figure 5 and Figure 6). Monitoring of the chromatograms at 240 nm did not reveal the presence of any other major compound, and this evidenced that the aforementioned phenolics were the major constituents (Figure S5).

However, significant differentiation was seen in the recovery yields, which were affected by both the type of treatment (hydrothermal/organosolv) and alkali catalyst (SoHy/SCar). As can be seen in Table 4, when either hydrothermal or organosolv treatment was accomplished without alkali catalyst, yields in both CouA and FA were particularly low, and did not exceed 0.49 mg of total hydroxycinnamates per g of dry CS mass. On the contrary, the SoHy-catalyzed hydrothermal treatment performed under optimized conditions (90 °C, 180 min) gave in total 31.79 mg of total hydroxycinnamates per g dry CS mass, showcasing the importance of the catalyst. The SCar-catalyzed treatment afforded 26.30 mg of total hydroxycinnamates per g dry CS mass and a lower CouA/FA ratio, indicating that the SoHy may be the more effective catalyst, favoring increased CouA recovery. On the other hand, the SoHy-catalyzed organosolv treatment (80 °C, 240 min) was the highest-performing system as it yielded 47.40 mg of total hydroxycinnamates per g dry CS mass (4.7 wt%), whereas the SCar-catalyzed treatment provided only 22.20 mg of total hydroxycinnamates per g dry CS mass.

By applying the SoHy-catalyzed organosolv treatment, 36.06 mg of CouA and 11.34 mg of FA could be retrieved from 1 g of CS biomass, which correspond to 3.61 and 1.13 wt%. A similar FA yield of about 1.1 wt% has been reported for SoHy-catalyzed corn bran treatment at 30 °C [31]. By contrast, CouA and FA yields of 2.8 and 1.1 wt%, respectively, were attained by treating corn straw under particularly harsh conditions, including 1 M SoHy, *T* = 100 °C, and *t* = 4 h [32]. Similarly, corn cobs treated with 2 M NaOH at 95 °C, for 30 min, afforded CouA and FA yields of 0.73 and 0.35 wt%, respectively [11]. Using SoHy-catalyzed ethanol organosolv treatments, other examinations achieved a total yield (CouA + FA) of 6 wt% [33], and CouA yield of 0.89 wt% [34]. On the other hand, corn fiber treatment with hydrolytic enzymes was shown to afford only 0.13 wt% FA retrieval [35], while SoHy-catalyzed ethanol organosolv treatment provided 0.85 wt% FA [36].

Considering the data given in Table 4, the hydrothermal treatments were in favor of obtaining higher CouA/FA ratios, compared to the organosolv ones. This phenomenon was manifested irrespective of the catalyst used, suggesting that the presence of ethanol might alleviate CouA recovery. Yet, the higher yields attained with the SoHy-catalyzed treatments indicated that catalyst strength was pivotal to recovering hydroxycinnamates from CS. Therefore, high recovery yields could be rather ascribed to the combination of ethanol/SoHy. This assumption is concurred by studies on sorghum stems [37] and cotton stalks [21], where ethanol addition promoted higher SoHy-catalyzed CouA recovery. The effect of the type of alcohol on polyphenol recovery has also been illustrated by studies on oat bran, where SoHy-catalyzed 1-propanol-organosolv treatment was significantly more efficacious for the recovery of both CouA and FA than the one performed with 2-propanol [25].

With regard to the higher performance of the treatments observed in the presence of SoHy compared to SCar, it would appear that effective hydrolysis of the esters bonds between hydroxycinnamates and polysaccharides (i.e., hemicellulose) required stronger catalysis. Because NaOH is strong and more reactive, it can more aggressively break lignin–hemicellulose linkages, disrupt the lignin–carbohydrate matrix, and liberate the associated hydroxycinnamates by cleaving ester linkages. On the other hand, SCar may provoke less damage to the lignin/polysaccharides complexes, which may result in lower removal of recalcitrant lignin/ester linkages, less structural disruption, and thus more limited hydroxycinnamate liberation.

In CS tissues, the lignin fraction is a polymeric matrix of covalently linked monolignols, which also contains high CouA and FA proportions [10,20]. Both these hydroxycinnamates occur in CS as integral constituents of the lignocellulosic network, linked with lignin and/or hemicellulose chains via various bonds. Almost 90% of CouA is attached to syringyl lignin moieties through ester bonds [38,39], while FA is principally esterified with arabinosyl residues of arabinoxylan chains. Nevertheless, FA may also act as a cross-link agent between lignin and arabinoxylan, being ether- or ester-linked onto lignin structures [10,40]. Appropriate combinations of heat, ethanol, and alkali catalyst can be crucial in hydroxycinnamate liberation, by assisting lignin/hemicellulose untangling, lignin dissolution, and finally lignin decomposition. All these processes may be facilitated in the presence of ethanol, which is involved in lignin fragmentation [26,41].

Both ether and ester bonds are prone to alkaline hydrolysis, and this enables the detachment of both CouA and FA from the lignocellulosic matrix, while ethanol enhances dissolution and entrainment into the liquid phase. Thus, ethanol-assisted lignin solubilization could further enhance hydroxycinnamate release. Additionally, ethanol may play a key role by permitting higher accessibility of ester bonds by sodium hydroxide, which is a rate-limiting step in the hydrolysis process [42]. Although ether-linked hydroxycinnamates may be cleaved mainly by acid catalysis, the ester-linked ones will be little affected. Ester bonds may be cleaved even under mild alkaline conditions, and the process may be accelerated by appropriate temperature regulation [42].

It has been reported that, during ethanol organosolv treatment, breakdown of *α*-aryl and *β*-aryl ether linkages may largely contribute to lignin decomposition [26,43]. Such reactions could further enhance hydroxycinnamate release, because they deconstruct large lignin polymers into smaller fractions, which in turn may buttress higher lignin solubilization. The relevant reactions may be accelerated by both acid and alkali catalysts, yet alkali-catalyzed treatments would significantly facilitate lignin solubilization [44], further enhanced by ethanol [45]. Hydroxycinnamate (FA) release has been strongly linked with alkali-solubilized lignin [42], and this might be another reason for the enhanced CouA and FA release under the condition used in this study. Therefore, alkali-catalyzed treatments may offer an advantage for increased hydroxycinnamate recovery.

It should also be emphasized that high temperatures may not always be in favor of obtaining higher hydroxycinnamate yields. For example, it has been demonstrated that treatment at 140 °C for more than 40 min was unsuitable, most probably provoking degradation of hydroxycinnamates, such as FA [31]. On the contrary, maximum FA recovery was achieved with hydrothermal treatment at 200 °C, with only 3.5 min resident time [27], while non-catalyzed ethanol organosolv treatment required 74 min at 160 °C, to yield maximum FA recovery [46]. On this ground, and considering that temperature and time are interdependent variables, appropriate adjustment of both variables to optimum levels would be of utmost importance to maximizing hydroxycinnamate recovery.

### 2.5. Antioxidant Effects

**Figure 7:**
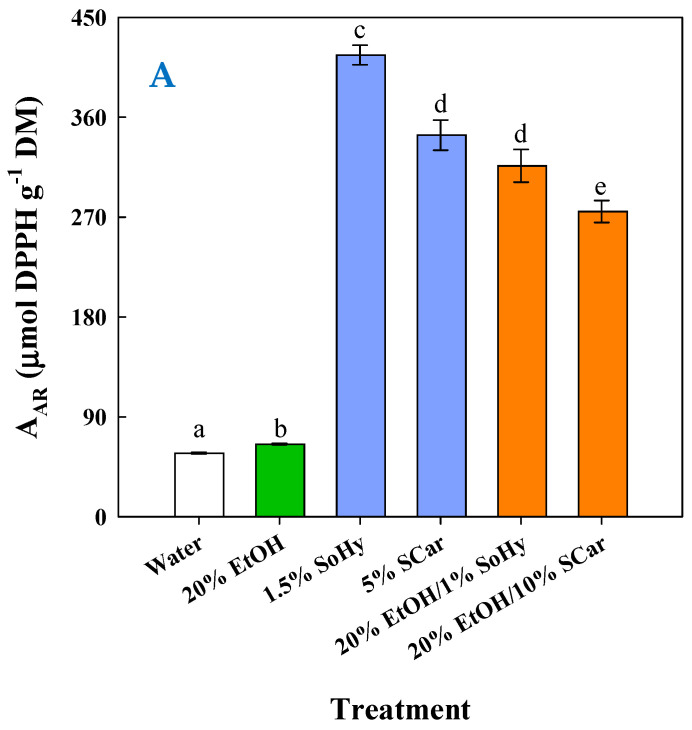

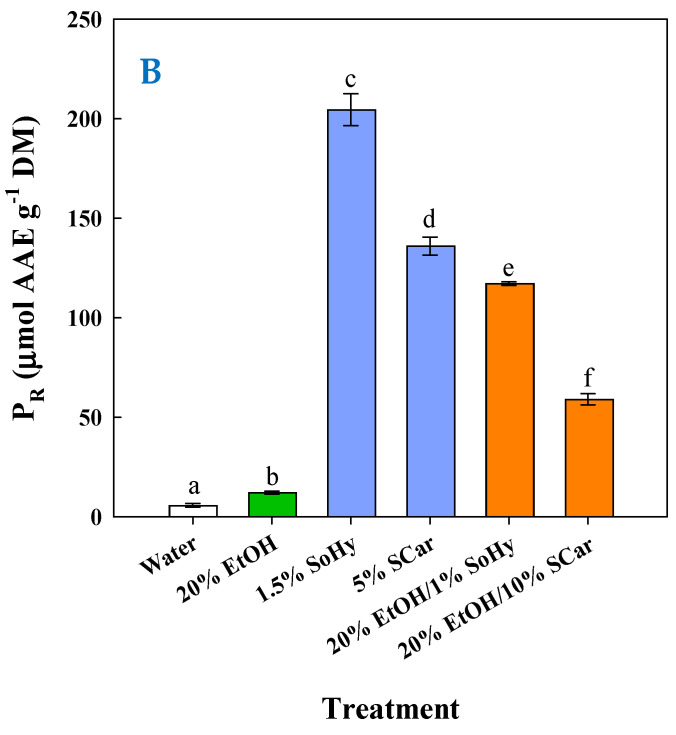
Measurement of the antiradical activity (**A**) and ferric-reducing power (**B**) of the extracts produced through the hydrothermal and organosolv treatments. SoHy and SCar denote the use of sodium hydroxide and sodium carbonate as the alkali catalyst, respectively. Bars represent means (n = 3) ± s.d. Bars assigned with different letters (a, b, c, d, e, and f) represent values with statistically significant difference (*p* < 0.05).

To have an insight into the impact of composition on the antioxidant behavior of the extracts generated, an appraisal was carried out by determining the antiradical activity (A_AR_) and the ferric-reducing power (P_R_). As can be seen in Figure 7A, the highest A_AR_ (*p* < 0.05) was found for the extract obtained by the SoHy-catalyzed hydrothermal treatment, followed by the extracts produced by the SCar-catalyzed hydrothermal treatment and the SoHy-catalyzed organosolv treatment. Likewise, the same order of activity was revealed when extracts were assessed for P_R_ (Figure 7B), which confirmed the results from the A_AR_ assay.

Considering that CouA and FA were the principal antioxidants in any extract tested, then it would be reasonably assumed that the antioxidant activity of the extracts was due to the presence of these polyphenolic compounds. Such effects have been demonstrated for a spectrum of plant tissues using various antioxidant assays, where the release of insoluble bound phenolics was shown to enhance antioxidant potency [47]. Furthermore, other studies have also indicated that the enhancement of the antioxidant activity in barley [48], barley bran [49], corn hydrolysate [50], and wheat bran [51] was directly proportional to the amount of phenolics liberated.

**Figure 8:**
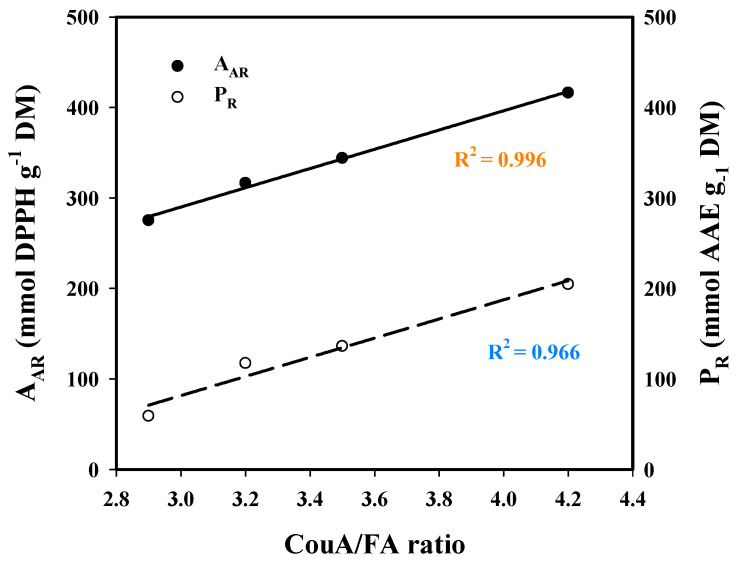
Plot showing the correlation between *p*-coumaric acid (CouA)/ferulic acid (FA) concentration ratio and the antiradical activity (A_AR_) and ferric-reducing power (P_R_), of the extracts generated through the hydrothermal and organosolv treatments.

Based on this evidence, it would be anticipated that both A_AR_ and P_R_ would correlate with the total polyphenol (CouA + FA) concentration, yet this was not observed. On the contrary, regression between the CouA/FA ratio and A_AR_ and P_R_ (Figure 8) clearly highlighted that the expression of antioxidant activity was strongly dictated by the relevant amounts of those hydroxycinnamates. On the other hand, it was rather surprising that strong antioxidant effects were exerted by the extracts having higher CouA proportion. This is because CouA has been demonstrated to display a much weaker antioxidant compared to FA in several studies [52,53,54]. On this basis, it could be argued that the antioxidant behavior observed might be attributed to synergistic and/or antagonistic effects, as suggested by earlier examinations on various polyphenol combinations [55,56,57]. Thus, the final outcome seen for both A_AR_ and P_R_ may reflect the manifestation of such effects.

## 3. Materials and Methods

### 3.1. Chemicals–Reagents

2,2-Diphenyl-1-picrylhydrazyl (DPPH) was purchased from Alfa Aesar (Karlsruhe, Germany) and 2,4,6-tris(2-pyridyl)-s-triazine (TPTZ) from Fluka (Steinheim, Germany). Procurement of sodium carbonate anhydrous was from Penta (Prague, Czechia), and L-ascorbic acid from Carlo Erba (Milano, Italy). Ferulic acid and *p*-coumaric acid were from Sigma-Aldrich (Steinheim, Germany). Iron chloride hexahydrate (FeCl_3_), citric acid anhydrous, and oxalic acid were purchased from Merck (Darmstadt, Germany). Absolute ethanol and Folin–Ciocalteu reagent were from Panreac (Barcelona, Spain). Solvents used for chromatography were of appropriate grade.

### 3.2. Corn Stover (CS) Collection and Processing

Collection of CS, which was composed essentially of dried residues of corn canes and leaves, was accomplished in October 2023, from a plantation neighboring the town of Karditsa (Central Greece). This crop residue was transferred to the laboratory and manually broken down to small segments; then, it was freeze dried for 24 h, comminuted in a laboratory mill, and sieved to collect a material with average particle size < 500 μm. This material was stored in air-tight containers, at 4 °C, for no longer than a week before use.

### 3.3. Hydrothermal and Organosolv Treatments

An exact aliquot of CS (2.5 g) was transferred into a 100 mL screw-cap glass vial, and 50 mL of either water (hydrothermal treatment) or hydroethanolic mixture (organosolv treatment), to give a liquid-to-solid ratio of 20 mL g^−1^ [21]. Both organosolv and hydrothermal treatments were performed with variable alkali (sodium hydroxide or sodium carbonate) catalyst concentrations, which were 5, 7.5, and 10% (*w*/*v*) for sodium carbonate, and 0.5, 1, and 1.5% for sodium hydroxide [22,23]. In the case of organosolv treatment, the ethanol proportion was always maintained at 20% (*v*/*v*), due to problems associated with sodium carbonate solubility [21]. For preliminary trials, hydrothermal treatments were performed for 300 min, at 90 °C. For the organosolv treatments, temperature was set at 80 °C, to avoid building up high vapor pressure due to ethanol. For the response surface methodology, both temperature and residence time values were defined by the experimental design. Both stirring and heating (at 400 rpm) were carried out by a temperature-controlled hotplate (Witeg, Wertheim, Germany). After each treatment, extracts were centrifuged at 10,000× *g* to remove solids, and the clear supernatant was used for all analytical determinations.

### 3.4. Polyphenol Recovery Kinetics

Kinetics of polyphenol recovery was traced by deploying first-order model, as previously described [58]:

Y_TP(*t*)_ = Y_TP(*s*)_(1 − *e*^−*kt*^) (1)

where Y_TP(*t*)_ corresponds to the total polyphenol yield at any time *t*, Y_TP(*s*)_ to the total polyphenol yield at saturation (equilibrium), and *k* to the first-order extraction rate constant (min^−1^). Sampling for total polyphenol measurements was accomplished at 10, 20, 30, 60, 120, 180, 240, and 300 min.

### 3.5. Estimation of Severity

Treatment severity was assessed using the alternative combined severity factor (CSF′) [28]:

CSF′ = *logR*_o_ + |pH − 7| (2)

The term *R*_o_ corresponds to the severity, as shown below [59]:

(3) $$R_{o}\;=\;t\;\times \;e^{\left(\frac{T-100}{14.75}\right)}$$

where 100 is the reference temperature (100 °C), and 14.75 is an empirical parameter related to the treatment temperature and activation energy.

### 3.6. Response Surface-Based Treatment Optimization

Treatment optimization was performed to detect the ideal settings of two instrumental factors, the residence time, *t*, and treatment temperature, *T*, on the performance of each treatment. The evaluation was based on the response values, the yield in total polyphenols (Y_TP_). These two independent variables (*t*, *T*) were chosen to set up a central composite experimental design, which encompassed 11 design points, with 3 of them being the central points. Three levels of codification, −1, 0, and 1, were used for both variables, and codification was performed as described previously [60]. Both codified and actual variable levels are displayed in Table 5.

Ranges for both variables were selected by appraising the kinetic data, yet data from recent works were also considered [21]. The significance of the mathematical models derived (R^2^, *p*), as well as the significance of each coefficient of the models, was assessed by appropriate statistical tests (lack-of-fit and analysis of variance—ANOVA tests), with 95% being the minimum level of significance.

### 3.7. Determination of Treatment Yield and Antioxidant Activity Measurements

The concentration of total polyphenol in the extracts was analyzed by employing a validated Folin–Ciocalteu protocol [61]. Gallic acid was the calibrating standard and results were given as gallic acid equivalents (GAEs). Total polyphenol yield (Y_TP_) was expressed as mg GAE per g of dried CS. Both antioxidant assays (the antiradical activity-A_AR_ and the ferric-reducing power-P_R_) were carried out deploying protocols published in full detail elsewhere [62]. A_AR_ were expressed as μmol DPPH per g of dried CS, and P_R_ as μmol ascorbic acid equivalents (AAE) per g of dried CS.

### 3.8. Chromatography

The liquid chromatography–diode array–tandem mass spectrometry (LC–DAD–MS/MS) set up and elution program used for the analysis have been described in detail elsewhere [25]. Mass spectra for tentative peak identification were obtained in negative ionization mode. For quantification, calibration curves for *p*-coumaric acid (R^2^ = 0.9982) and ferulic acid (R^2^ = 0.9980) were prepared using commercial standards (0–50 μg mL^−1^). These standard solutions were freshly made in HPLC-grade methanol just before the analysis.

### 3.9. Statistics and Data Processing

Treatments (hydrothermal, organosolv) were accomplished at least twice, while chromatographic and spectrophotometric analyses were performed in triplicate. The values presented are average ± standard deviation (SD). The JMP™ Pro 16 software (SAS, Cary, NC, USA) was employed for the experimental design of the response surface methodology, and for computing statistical tests (ANOVA, lack-of-fit). Similarly, SigmaPlot™ 15.0 (Systat Software Inc., San Jose, CA, USA) was employed for non-linear and linear regressions, performed at least at a 95% significance level. Considering that the data obtained from the screening, antioxidant testing, and chromatographic examinations did not display normal distribution, as revealed by the Shapiro–Wilk test, statistically significant differences were detected using the Kruskal–Wallis test, performed by IBM SPSS Statistics™ 29 (SPSS Inc., Chicago, IL, USA).

## 4. Conclusions

This work had as its objective to establish a high-performance process for boosting the recovery of antioxidant hydroxycinnamates from CS. The SoHy-catalyzed ethanol organosolv treatment was demonstrated to be more efficient compared to the hydrothermal one, while SoHy outperformed SCar in catalyzing hydroxycinnamate liberation from the lignocellulosic matrix. The SoHy-catalyzed organosolv treatment resulted in significantly higher recoveries for both *p*-coumaric and ferulic acids and, under optimized settings of temperature and time determined through response surface methodology, the yield in total hydroxycinnamates (*p*-coumaric acid + ferulic acid) was about 4.7 wt%. This level was achieved with a relatively low severity treatment, which might be of importance in establishing integrated biorefinery processes, with the prospect of producing high value-added phytochemicals. These compounds, owing to their multifaceted biological activities, could be of wide applicability to food, cosmetics, and pharmaceutical manufacturing. The green character of the process could be significantly enhanced by replacing the corrosive SoHy by the benign SCar, yet issues pertaining to compromising recovery yields should be resolved. The methodology proposed is simple, relatively benign, and straight-forward, using a highly abundant and low-cost bioresidue as the resource and inexpensive chemicals as catalysts. Furthermore, purification of the extracts generated to produce hydroxycinnamate-enriched extracts would be rather simple using, e.g., chromatographic or liquid–liquid solvent extraction techniques, due to the high concentration of the extracts in the target compounds. The remaining biomass could serve as a source to recover other valuable substances, such as lignin and cellulose/hemicellulose. Thus, it could be supported that such an approach presented in this investigation may be directly applicable on an industrial scale, with the appropriate scale-up and feasibility studies provided. It is to be emphasized that this work compared for the first time two methodologies (hydrothermal vs. organosolv) and two alkali catalysts (SoHy vs. SCar) for the high-performance production of hydroxycinnamate-enriched extracts from CS. In addition, it is the first time that such a high yield in total hydroxycinnamates using CS as the source is reported. Further treatment optimization is currently under way and it is anticipated to enhance treatment performance for establishing a highly efficient and fully sustainable process. This will also bring out further the potential of CS as an exceptionally rich lignocellulosic bioresource of precious biocompounds.

## Figures and Tables

**Table 1:** Kinetic data and yield in total polyphenols (Y_TP_) obtained by performing hydrothermal and organosolv treatments to corn stover.

Treatment	*T* (°C)	R^2^ *	*k* (min^−1^) × 10^−3^	Y_TP(*s*)_ (mg GAE g^−1^ DM)
Hydrothermal				
1.5% SoHy	50	0.99	93.9	27.9 ± 1.9 ^a^
	70	0.95	87.4	55.1 ± 3.2 ^b^
	90	1.00	39.6	73.1 ± 5.2 ^c^
5% SCar	50	0.93	26.9	13.4 ± 0.8 ^d^
	70	0.92	20.1	20.7 ± 1.0 ^e^
	90	0.97	70.9	50.4 ± 3.6 ^b^
Organosolv				
1% SoHy	40	0.95	39.3	23.2 ± 1.7 ^f^
	60	0.94	38.7	30.4 ± 2.1 ^g^
	80	0.94	20.3	59.4 ± 3.9 ^h^
10% SCar	40	0.91	30.1	16.8 ± 0.9 ^i^
	60	0.93	27.1	19.5 ± 1.0 ^e^
	80	0.97	25.4	44.7 ± 2.8 ^j^

* Indicates correlation of the kinetic model fitting to the experimental data. Letters a–j denote statistically significant difference (*p* < 0.05).

**Table 2:** Response surface-derived models describing the effect of treatment variables (X_1_, X_2_) on the yield in total polyphenols (Y_TP_).

Treatment	Equation (Model)	R^2^	*p*
Hydrothermal			
1.5% SoHy	Y_TP_ = 61.6 + 22.3X_1_ − 9.7X_1_^2^	1.00	0.0001
5% SCar	Y_TP_ = 22.1 + 20X_1_ + 1.9X_2_ + 11.5X_1_^2^	1.00	0.0001
Organosolv			
1% SoHy	Y_TP_ = 33.6 + 14.2X_1_ + 6.8X_2_ + 5.8X_1_X_2_	0.97	0.0006
10% SCar	Y_TP_ = 18.7 + 13.4X_1_ + 3.1X_2_ + 10.9X_1_^2^	1.00	<0.0001

**Table 3:** Data on optimum treatment conditions and maximum total polyphenol yield, as predicted by the models built through response surface methodology. The associated severity, given as combined severity factor (CSF′), is also provided.

Treatment	Optimal *T* (°C)	Optimal *t* (min)	Maximum Predicted Y_TP_ (mg GAE g^−1^ DM)	Severity (CSF′)
Hydrothermal (1.5% SoHy)	90	180	74.4 ± 3.6 ^a^	8.19
Hydrothermal (5% SCar)	90	300	57.1 ± 3.7 ^b^	6.66
Organosolv (20% EtOH/1% SoHy)	80	240	64.2 ± 6.8 ^a^	7.64
Organosolv (20% EtOH/10% SCar)	80	240	47.8 ± 1.8 ^c^	6.55

Letters a, b, and c denote statistically significant difference (*p* < 0.05).

**Table 4:** Analytical data pertaining to polyphenolic composition of extracts obtained by deploying hydrothermal and organosolv treatments. SoHy and SCar denote the use of sodium hydroxide and sodium carbonate as alkali catalysts, respectively.

Treatment	Y (mg g^−1^ DM)			
	CouA	FA	Ratio CouA/FA	Total
Water	0.34 ± 0.01 ^a^	0.15 ± 0.01 ^a^	2.3	0.49
Hydrothermal (1.5% SoHy)	25.64 ± 0.5 ^b^	6.15 ± 0.19 ^b^	4.2	31.79
Hydrothermal (5% SCar)	20.48 ± 0.94 ^c^	5.82 ± 0.23 ^b,e^	3.5	26.30
20% EtOH	0.31 ± 0.03 ^a^	0.08 ± 0.01 ^c^	3.9	0.39
Organosolv (20% EtOH/1% SoHy)	36.06 ± 1.20 ^d^	11.34 ± 0.82 ^d^	3.2	47.40
Organosolv (20% EtOH/10% SCar)	16.51 ± 0.20 ^e^	5.69 ± 0.17 ^e^	2.9	22.20

Values assigned with different letters (a, b, c, d, and e) within columns denote statistically significant difference (*p* < 0.05).

**Table 5:** The variables used in the response surface methodology, and their codified and actual levels.

Process Variables	Codes	Coded Variable Level		
		−1	0	1
*t* (min)	X_1_	60	180	300
*T* (°C) (hydrothermal treatment)	X_2_	50	70	90
*T* (°C) (organosolv treatment)		40	60	80

## Data Availability

The original contributions presented in this study are included in the article/Supplementary Material. Further inquiries can be directed to the corresponding authors.

## Supplementary Materials

The following supporting information can be downloaded at https://www.mdpi.com/article/10.3390/molecules30214297/s1, Figure S1: Optimization of the hydrothermal treatment with sodium hydroxide as catalyst. Figure S2: Optimization of the hydrothermal treatment with sodium carbonate as catalyst. Figure S3: Optimization of the organosolv treatment with sodium hydroxide as catalyst. Figure S4: Optimization of the organosolv treatment with sodium carbonate as catalyst. Figure S5: Chromatogram of CS extracts obtained using organosolv treatment with SoHy as catalyst. Table S1: Data illustrating the combination of the hydrothermal treatment variables (*t*, *T*) employed for the experimental design, the actual response (Y_TP_) values, and the predicted values determined by the models derived from the response surface methodology. Table S2: Data illustrating the combination of the hydrothermal treatment variables (*t*, *T*) employed for the experimental design, the actual response (Y_TP_) values, and the predicted values determined by the models derived from the response surface methodology. Table S3: Data illustrating the combination of the organosolv treatment variables (*t*, *T*) employed for the experimental design, the actual response (Y_TP_) values, and the predicted values determined by the models derived from the response surface methodology. Table S4: Data illustrating the combination of the organosolv treatment variables (*t*, *T*) employed for the experimental design, the actual response (Y_TP_) values, and the predicted values determined by the models derived from the response surface methodology.
