# Supplementary material for: Hydrothermal and Organosolv Treatments for Hydroxycinnamate Release from Corn Stover: Strong versus Mild Alkaline Catalysis
Source: Molecules. 2025 Nov 5;30(21):4297. doi: 10.3390/molecules30214297 (PMC12609553; doi:10.3390/molecules30214297)
Supplement: Supplementary file 1 [file molecules-30-04297-s001.zip › molecules-3920493-supplementary.pdf]

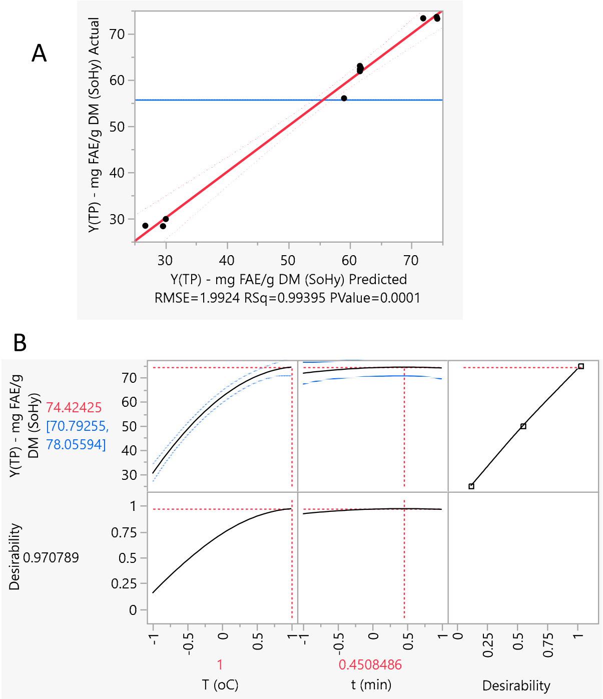

Lack Of Fit

Source	DF	Sum of Squares	Mean Square	F Ratio
Lack Of Fit	3	19.177210	6.39240	19.0345
Pure Error	2	0.671667	0.33583	**Prob > F**
Total Error	5	19.848877		0.0503
				**Max RSq**
				0.9998

Parameter Estimates

Term	Estimate	Std Error	t Ratio	Prob>|t|
Intercept	61.613684	1.022095	60.28	0.000000
T (oC)	22.26	0.813406	27.37	0.000001
t (min)	1.3733333	0.813406	1.69	0.152140
T (oC)*t (min)	-0.2875	0.996215	-0.29	0.784475
T (oC)*T (oC)	-9.694211	1.251805	-7.74	0.000574
t (min)*t (min)	-1.204211	1.251805	-0.96	0.380241

Effect Tests

Source	Nparm	DF	Sum of Squares	F Ratio	Prob > F
T (oC)	1	1	2973.0456	748.9204	0.000001
t (min)	1	1	11.3163	2.8506	0.152140
T (oC)*t (min)	1	1	0.3306	0.0833	0.784475
T (oC)*T (oC)	1	1	238.0769	59.9724	0.000574
t (min)*t (min)	1	1	3.6736	0.9254	0.380241

Figure S1: Optimization of the hydrothermal treatment with sodium hydroxide as catalyst. Diagram (A) shows the correlation between the predicted and actual values of the response (Y_{TP}), obtained after implementing response surface methodology. The square correlation coefficient (R^2) and the *p*-value for the model are also given. Diagram (B) shows the desirability factor, the maximum predicted Y_{TP}, as well as the theoretical optimum *t* and *T*. The inset tables contain the statistics associated with the response surface methodology. Values denoted with asterisk are statistically significant ($p < 0.05$).

A

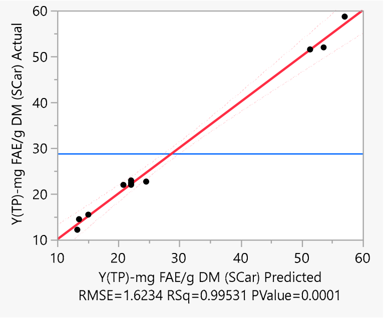

Lack Of Fit				
Source	DF	Sum of Squares	Mean Square	F Ratio
Lack Of Fit	3	12.730736	4.24358	18.9784
Pure Error	2	0.447200	0.22360	Prob > F
Total Error	5	13.177936		0.0505
				Max RSq
				0.9998

Parameter Estimates				
Term	Estimate	Std Error	t Ratio	Prob>|t|
Intercept	22.105263	0.832812	26.54	0.000001
T (oC)	20.008333	0.66277	30.19	0.000001
t (min)	1.8633333	0.66277	2.81	0.037485
T (oC)*t (min)	0.9675	0.811725	1.19	0.286780
T (oC)*T (oC)	11.501842	1.019982	11.28	0.000096
t (min)*t (min)	0.6068421	1.019982	0.59	0.577775

B

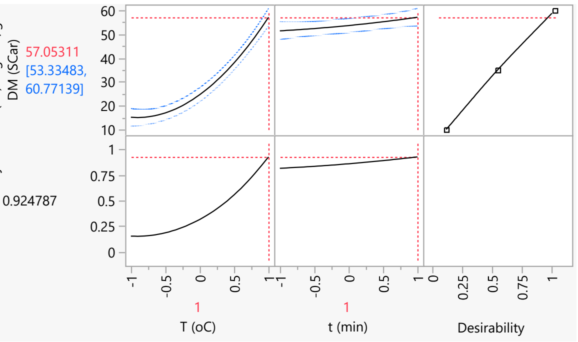

Effect Tests					
Source	Nparm	DF	Sum of Squares	F Ratio	Prob > F
T (oC)	1	1	2402.0004	911.3720	0.000001
t (min)	1	1	20.8321	7.9041	0.037485
T (oC)*t (min)	1	1	3.7442	1.4206	0.286780
T (oC)*T (oC)	1	1	335.1407	127.1598	0.000096
t (min)*t (min)	1	1	0.9329	0.3540	0.577775

Figure S2: Optimization of the hydrothermal treatment with sodium carbonate as catalyst. Diagram (A) shows the correlation between the predicted and actual values of the response (Y_{TP}), obtained after implementing response surface methodology. The square correlation coefficient (R^2) and the *p*-value for the model are also given. Diagram (B) shows the desirability factor, the maximum predicted Y_{TP}, as well as the theoretical optimum *t* and *T*. The inset tables contain the statistics associated with the response surface methodology. Values denoted with asterisk are statistically significant (red: $p < 0.05$; orange, $p < 0.001$).

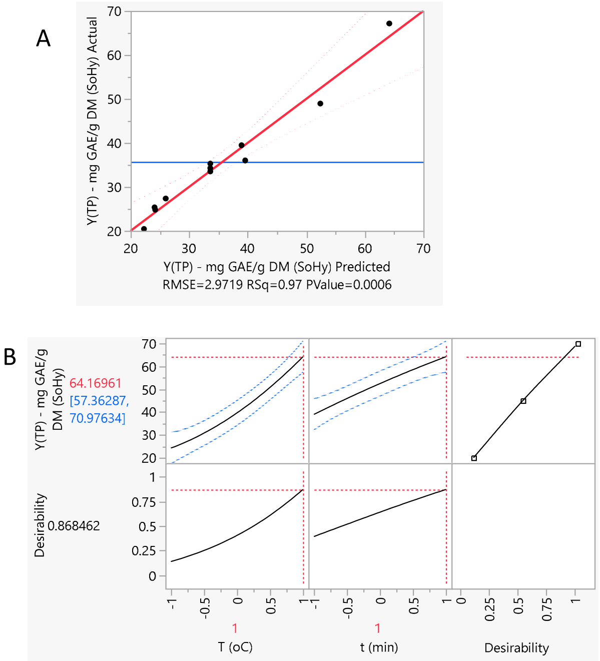

Lack Of Fit

Source	DF	Sum of Squares	Mean Square	F Ratio
Lack Of Fit	3	42.531621	14.1772	17.3996
Pure Error	2	1.629600	0.8148	**Prob > F**
Total Error	5	44.161221		0.0548
				**Max RSq**
				0.9990

Parameter Estimates

Term	Estimate	Std Error	t Ratio	Prob>|t|
Intercept	33.619474	1.524557	22.05	0.000004
T (oC)	14.153333	1.213277	11.67	0.000081
t (min)	6.7816667	1.213277	5.59	0.002529
T (oC)*t (min)	5.8275	1.485955	3.92	0.011163
T (oC)*T (oC)	4.6263158	1.867194	2.48	0.056000
t (min)*t (min)	-0.838684	1.867194	-0.45	0.672110

Effect Tests

Source	Nparm	DF	Sum of Squares	F Ratio	Prob > F
T (oC)	1	1	1201.9011	136.0811	0.000081
t (min)	1	1	275.9460	31.2430	0.002529
T (oC)*t (min)	1	1	135.8390	15.3799	0.011163
T (oC)*T (oC)	1	1	54.2204	6.1389	0.056000
t (min)*t (min)	1	1	1.7819	0.2018	0.672110

Figure S3: Optimization of the organosolv treatment with sodium hydroxide as catalyst. Diagram (A) shows the correlation between the predicted and actual values of the response (Y_{TP}), obtained after implementing response surface methodology. The square correlation coefficient (R^2) and the *p*-value for the model are also given. Diagram (B) shows the desirability factor, the maximum predicted Y_{TP}, as well as the theoretical optimum *t* and *T*. The inset tables contain the statistics associated with the response surface methodology. Values denoted with asterisk are statistically significant (red: $p < 0.05$; orange, $p < 0.001$).

A

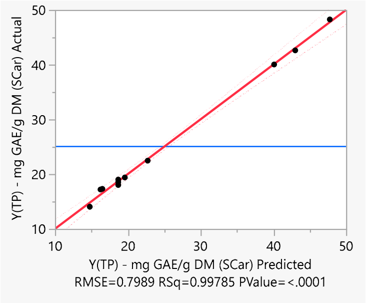

Lack Of Fit				
Source	DF	Sum of Squares	Mean Square	F Ratio
Lack Of Fit	3	2.6894614	0.896487	3.5740
Pure Error	2	0.5016667	0.250833	Prob > F
Total Error	5	3.1911281		0.2263
				Max RSq
				0.9997

Parameter Estimates

Term	Estimate	Std Error	t Ratio	Prob>|t|
Intercept	18.695263	0.409822	45.62	0.000000
T (oC)	13.378333	0.326146	41.02	0.000000
t (min)	3.12	0.326146	9.57	0.000211
T (oC)*t (min)	0.715	0.399445	1.79	0.133468
T (oC)*T (oC)	10.941842	0.501927	21.80	0.000004
t (min)*t (min)	0.9268421	0.501927	1.85	0.124095

B

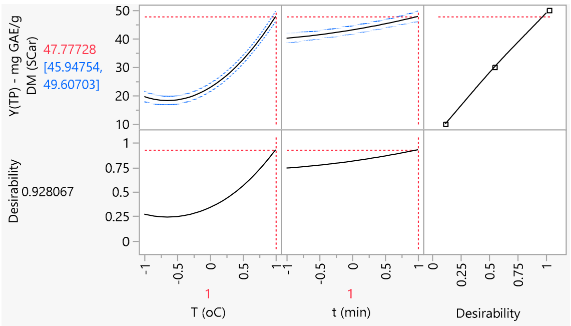

Effect Tests

Source	Nparm	DF	Sum of Squares	F Ratio	Prob > F
T (oC)	1	1	1073.8788	1682.601	0.000000
t (min)	1	1	58.4064	91.5137	0.000211
T (oC)*t (min)	1	1	2.0449	3.2040	0.133468
T (oC)*T (oC)	1	1	303.3006	475.2247	0.000004
t (min)*t (min)	1	1	2.1762	3.4098	0.124095

Figure S4: Optimization of the organosolv treatment with sodium carbonate as catalyst. Diagram (A) shows the correlation between the predicted and actual values of the response (Y_{TP}), obtained after implementing response surface methodology. The square correlation coefficient (R^2) and the *p*-value for the model are also given. Diagram (B) shows the desirability factor, the maximum predicted Y_{TP}, as well as the theoretical optimum *t* and *T*. The inset tables contain the statistics associated with the response surface methodology. Values denoted with asterisk are statistically significant ($p < 0.05$).

Table S1: Data illustrating the combination of the hydrothermal treatment variables (*t*, *T*) employed for the experimental design, the actual response (Y_{TP}) values, and the predicted values determined by the models derived from the response surface methodology. The catalyst used was sodium hydroxide.

**Design point**	**Independent variables**		**Responses**	
			**Y_{TP} (mg GAE g^{-1} dw)**	
	**X_1 (*T*, °C)**	**X_2 (*t*, min)**	**Measured**	**Predicted**
1	-1 (50)	-1 (60)	28.4	26.8
2	-1 (50)	1 (300)	29.8	30.1
3	1 (90)	-1 (60)	73.3	71.9
4	1 (90)	1 (300)	73.6	74.1
5	-1 (50)	0 (180)	28.3	29.7
6	1 (90)	0 (180)	73.2	74.2
7	0 (70)	-1 (60)	56.0	59.0
8	0 (70)	1 (300)	62.5	61.8
9	0 (70)	0 (180)	62.3	61.6
10	0 (70)	0 (180)	61.8	61.6
11	0 (70)	0 (180)	63.0	61.6

Table S2: Data illustrating the combination of the hydrothermal treatment variables (*t*, *T*) employed for the experimental design, the actual response (Y_{TP}) values, and the predicted values determined by the models derived from the response surface methodology. The catalyst used was sodium carbonate.

Design point	Independent variables		Responses	
			Y_{TP} (mg GAE g^{-1} dw)	
	X_1 (T, °C)	X_2 (t, min)	Measured	Predicted
1	-1 (50)	-1 (60)	12.1	13.3
2	-1 (50)	1 (300)	15.4	15.1
3	1 (90)	-1 (60)	51.5	51.4
4	1 (90)	1 (300)	58.6	57.1
5	-1 (50)	0 (180)	14.4	13.6
6	1 (90)	0 (180)	51.9	53.6
7	0 (70)	-1 (60)	21.9	20.8
8	0 (70)	1 (300)	22.6	24.6
9	0 (70)	0 (180)	22.3	22.1
10	0 (70)	0 (180)	22.9	22.1
11	0 (70)	0 (180)	21.9	22.1

Table S3: Data illustrating the combination of the organosolv treatment variables (*t*, *T*) employed for the experimental design, the actual response (Y_{TP}) values, and the predicted values determined by the models derived from the response surface methodology. The catalyst used was sodium hydroxide.

**Design point**	**Independent variables**		**Responses**	
			**Y_{TP} (mg GAE g^{-1} dw)**	
	**X_1 (T, °C)**	**X_2 (t, min)**	**Measured**	**Predicted**
1	-1 (40)	-1 (60)	20.4	22.3
2	-1 (40)	1 (300)	24.8	24.2
3	1 (80)	-1 (60)	39.5	39.0
4	1 (80)	1 (300)	67.1	64.2
5	-1 (40)	0 (180)	25.3	24.1
6	1 (80)	0 (180)	48.9	52.4
7	0 (60)	-1 (60)	27.3	26.0
8	0 (60)	1 (300)	36.0	39.6
9	0 (60)	0 (180)	34.3	33.6
10	0 (60)	0 (180)	33.5	33.6
11	0 (60)	0 (180)	35.3	33.6

Table S4: Data illustrating the combination of the organosolv treatment variables (*t*, *T*) employed for the experimental design, the actual response (Y_{TP}) values, and the predicted values determined by the models derived from the response surface methodology. The catalyst used was .

**Design point**	**Independent variables**		**Responses**	
			**Y_{TP} (mg GAE g^{-1} dw)**	
	**X_1 (*T*, °C)**	**X_2 (*t*, min)**	**Measured**	**Predicted**
1	-1 (40)	-1 (60)	14.0	14.8
2	-1 (40)	1 (300)	19.4	19.6
3	1 (80)	-1 (60)	40.0	40.1
4	1 (80)	1 (300)	48.3	47.8
5	-1 (40)	0 (180)	17.2	16.3
6	1 (80)	0 (180)	42.6	43.0
7	0 (60)	-1 (60)	17.3	16.5
8	0 (60)	1 (300)	22.5	22.7
9	0 (60)	0 (180)	18.6	18.7
10	0 (60)	0 (180)	18.0	18.7
11	0 (60)	0 (180)	19.0	18.7

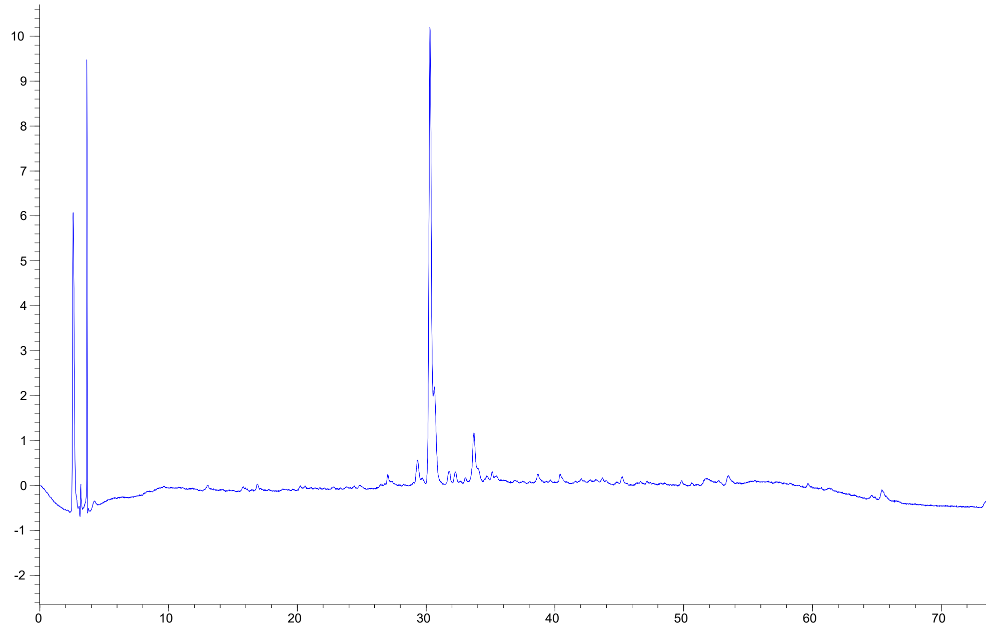

Figure S5: Chromatogram of CS extracts obtained using organosolv treatment with SoHy as catalyst. The chromatogram was monitored at 240 nm.
